# Description and first insights on a large genomic biobank of lung transplantation

**DOI:** 10.1038/s41431-024-01683-y

**Published:** 2024-08-20

**Authors:** Simon Brocard, Martin Morin, Nayane dos Santos Brito Silva, Benjamin Renaud-Picard, Benjamin Coiffard, Xavier Demant, Loïc Falque, Jérome Le Pavec, Antoine Roux, Thomas Villeneuve, Christiane Knoop, Jean-François Mornex, Mathilde Salpin, Véronique Boussaud, Olivia Rousseau, Vincent Mauduit, Axelle Durand, Antoine Magnan, Pierre-Antoine Gourraud, Nicolas Vince, Mario Südholt, Adrien Tissot, Sophie Limou, COLT consortium, Benjamin Renaud-Picard, Benjamin Coiffard, Xavier Demant, Loïc Falque, Antoine Roux, Thomas Villeneuve, Christiane Knoop, Jean-François Mornex, Mathilde Salpin, Véronique Boussaud, Antoine Magnan, Adrien Tissot, Jérome Le Pavec

**Affiliations:** 1 Nantes Université, CHU Nantes, Centrale Nantes, Inserm, Center for Research in Transplantation and Translational Immunology, UMR 1064, ITUN, Nantes, France; 2 IMT Atlantique - DAPI - Département Automatique, Productique et Informatique, Nantes, France; 3 São Paulo State University, Molecular Genetics and Bioinformatics Laboratory, School of Medicine, Botucatu, State of São Paulo Brazil; 4 Department of Respiratory Medicine and Strasbourg Lung Transplant Program, Hôpitaux Universitaires de Strasbourg, Strasbourg, France; Université de Strasbourg, Inserm UMR 1260, Strasbourg, France; 5 Aix Marseille Univ, Department of Respiratory Medicine and Lung Transplantation, APHM, Hôpital Nord, Marseille, France; 6 Service de Pneumologie, Centre Hospitalier Universitaire de Bordeaux, Pessac, France; 7 Service Hospitalier Universitaire de Pneumologie et Physiologie, CHU Grenoble Alpes, Pôle Thorax et Vaisseaux, Grenoble, France; 8 Service de Pneumologie et Transplantation Pulmonaire, Groupe hospitalier Marie-Lannelongue -Saint Joseph, Le Plessis-Robinson, Université Paris-Saclay, Le Kremlin Bicêtre, UMR_S 999, Université Paris–Sud, INSERM France, Paris, France; 9 Pneumology, Adult Cystic Fibrosis Center and Lung Transplantation Department Hôpital Foch, Suresnes, Université de Versailles Saint Quentin Paris-Saclay, INRAe UMR 0892, Paris Transplant Group, Paris, France; 10 CHU Toulouse, Service de Pneumologie, Université Toulouse III-Paul Sabatier, Toulouse, France; 11 Service de Pneumologie, CHU Erasme, Bruxelles, Belgium; 12 Université de Lyon, Université Lyon 1, PSL, EPHE, INRAE, IVPC, hospices civils de Lyon, groupement hospitalier est, service de pneumologie, Orphalung, RESPIFIL Lyon, Lyon, France; 13 APHP Nord-Université Paris Cité, Hôpital Bichat, Service de Pneumologie B et Transplantation Pulmonaire, Université Paris Cité, PHERE UMRS 1152, Paris, France; 14 APHP, Service de Pneumologie, Hôpital Cochin, Paris, France; 15 Hôpital Foch, Université de Versailles Saint Quentin Paris-Saclay, INRAe UMR 0892, Paris, France; 16 LS2N - STACK - Software Stack for Massively Geo-Distributed Infrastructures, Nantes, France; 17 CHU Nantes, Nantes Université, Service de Pneumologie, Nantes, France

**Keywords:** Genome-wide association studies, Immunogenetics, Biomarkers, Genome informatics

## Abstract

The main limitation to long-term lung transplant (LT) survival is chronic lung allograft dysfunction (CLAD), which leads to irreversible lung damage and significant mortality. Individual factors can impact CLAD, but no large genetic investigation has been conducted to date. We established the multicentric Genetic COhort in Lung Transplantation (GenCOLT) biobank from a rich and homogeneous sub-part of COLT cohort. GenCOLT collected DNA, high-quality GWAS (genome-wide association study) genotyping and robust *HLA* data for donors and recipients to supplement COLT clinical data. GenCOLT closely mirrors the global COLT cohort without significant variations in variables like demographics, initial disease and survival rates (*P* > 0.05). The GenCOLT donors were 45 years-old on average, 44% women, and primarily died of stroke (54%). The recipients were 48 years-old at transplantation on average, 45% women, and the main underlying disease was chronic obstructive pulmonary disease (45%). The mean follow-up time was 67 months and survival at 5 years was 57.3% for the CLAD subgroup and 97.4% for the non-CLAD subgroup. After stringent quality controls, GenCOLT gathered more than 7.3 million SNP and HLA genotypes for 387 LT pairs, including 91% pairs composed of donor and recipient of European ancestry. Overall, GenCOLT is an accurate snapshot of LT clinical practice in France and Belgium between 2009 and 2018. It currently represents one of the largest genetic biobanks dedicated to LT with data available simultaneously for donors and recipients. This unique cohort will empower to run comprehensive GWAS investigations of CLAD and other LT outcomes.

## Introduction

According to the international registry of the International Society of Heart and Lung Transplantation (ISHLT), the number of lung transplants (LT) performed each year worldwide has gradually increased over the past decades, from about 250 in 1990 to more than 4000 today [1]. In France, 334 lung transplantations were performed in 2022 alone [2, 3]. Despite improvements in surgical techniques [4–7] and immunosuppressive treatment management [8], the long-term outcome remains limited with a mean survival of 63% at 5 years post-transplantation in Europe [9]. The main limitation to long-term survival is the development of chronic lung allograft dysfunction (CLAD) [10, 11], which is defined by a persistent decline (≥ 20%) of the FEV1 (forced expiratory volume in 1 s) value from baseline.

Several risk factors for CLAD have been suggested including alloimmune factors (cellular or antibody-mediated rejections) and non-alloimmune factors (primary graft dysfunction, pulmonary infection, airway pollution or gastro-esophageal reflux disease) [11]. Indubitably, individual factors, either recipient or donor related, play a role in the biological responses of lung injury and in the end, in the occurrence of CLAD [12]. To date, no extensive genetic research has been conducted to explore the potential influence of genetic factors on chronic lung allograft dysfunction. In contrast, several genomic initiatives have been conducted in kidney transplantation [13] and confirmed the importance of *HLA* mismatches, as well as an emerging role for non-HLA factors and mismatches. In LT, the investigation of *HLA* mismatches has yielded conflicting results [14], but more recent data suggests a link between *HLA* mismatches and an increased risk for bronchiolitis obliterans syndrome (BOS) [15] and CLAD [16]. Given this knowledge gap, we believe it will be highly valuable to develop large-scale genetic biobanks and genomic analyses to identify associations between genotypes and LT-related outcomes.

Here, we describe the clinical and genetic characteristics of GenCOLT which gathers DNA samples, GWAS (genome-wide association study) data and clinical records for 392 donor-recipient pairs (*n* = 784 individuals) from 11 French and Belgian clinical centers.

## Material and methods

### Description of the COLT clinical database

GenCOLT was built as a sub-part of the Cohort in Lung Transplantation (COLT – NCT00980967), whose main focus was the discovery of CLAD risk factors [16, 17]. The ethics committee (*Comité de Protection des Personnes*, 2009-A00036-51) approved the study and all participants provided a written informed consent (CNIL French data protection authority, #911142). COLT was a prospective study, which included patients from twelve clinical centers between September 2009 to December 2018: CHU Nantes, Hospices Civils de Lyon, Assistance Publique-Hôpitaux de Marseille, Hôpital Foch (Paris), Hôpital Européen Georges Pompidou (Paris), CHU Grenoble, CHRU Strasbourg, CHU Bordeaux, Hôpital Bichat (Paris), Hôpital Marie Lannelongue (Paris area) and CHU Toulouse from France, as well as the Erasme Hospital (Brussels, Belgium). COLT comprises a total of 1413 transplanted patients from whom clinical data and biological samples were collected. The clinical records and follow-up data from each participating center are centralized in a secured online database coordinated by the Nantes University Hospital. According to protocol, follow-up visits were conducted at 1-month and 6-month after transplantation, and then every 6 months for a period of up to 5 years for biological sample (blood) collection, and up to 10 years for clinical data. Clinical follow-up data include functional data such as allograft dysfunction, infection, and pulmonary function test. In addition, every relevant clinical event occurring and all pulmonary function tests performed between two protocol visits (e.g. infection, acute cellular rejection, hospital visit) were recorded in the database and considered by the adjudication committee which includes respiratory physicians from at least five different centers.

### Clinical phenotypes

All COLT patients underwent individual phenotyping by an adjudication committee that gathered at least 5 investigator physicians from the different participating centres. Pulmonary function tests (last FEV1 values before rejection prognostic are assessed to correlate a degradation of at least 20% FEV1 to a chronic graft dysfunction), relevant chest computed tomography and medical history, especially potential confounding factors, were reviewed for a collegial decision on phenotype initially based on the 2014 proposed classification and then on the 2019 ISHLT consensus report on CLAD [17]. Recipients were classified as follows: BOS, restrictive allograft syndrome (RAS), azithromycin responsive allograft dysfunction (ARAD), non-CLAD (alive 2 years after transplant without CLAD) and other (death within 3 months after transplantation, death without CLAD, insufficient data to conclude or confounding factors).

### Description of the GenCOLT genetic data

#### DNA collection and GWAS genotyping

GenCOLT is a DNA biobank that was established as an extension of COLT. At this stage, GenCOLT has gathered DNA samples from 392 pairs of LT donors and recipients (*n* = 784 individuals) across 12 centers. We included all adult patients (age ≥18 years-old) with a survival over 3 months post-transplantation, for whom consent for genetic investigations and a DNA sample were available for both the donor and the recipient. The collection of samples from deceased donors for scientific research has been authorized by the *Agence de Biomédecine* (PFS09-003). A protocol has been put in place, which includes researching whether or not the donor is opposed to the use of his organs or body parts for research and information for relatives. The GenCOLT cohort is located and managed by the *Centre des Ressources Biologiques* at the Nantes University Hospital.

Each DNA sample was assessed for volume, concentration, purity (260/230 and 260/280 absorbance ratios) and degradation by analyzing migration on an agarose gel. Following the manufacturer recommendations, the DNA samples were normalized to a volume of 20 µL and a concentration of 10 ng/µL. They were randomized based on sex and donor/recipient status in 96-well plates to minimize batch effects. Subsequently, the DNA samples underwent genotyping using the Axiom PMRA (Precision Medicine Research Array) chips (ThermoFisher, Waltham MA, USA), which cover 902,560 genetic variants (or SNPs, single nucleotide polymorphisms) including those found in the *HLA* and *KIR* polymorphic genomic regions, as well as other relevant genes for research in cancer and immunology. We followed the Axiom 2.0 Thermofisher standardized protocols and guidelines during the genotyping process.

#### Data processing and imputations

**Fig. 1:**
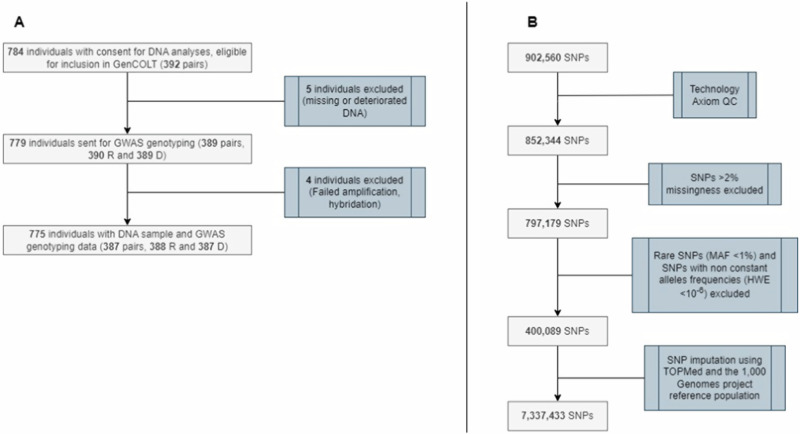
Building the GenCOLT biobank and robust GWAS SNP data. **A** Establishment of the GenCOLT DNA cohort. Initially, a total of 784 individuals were selected from the COLT biobank for DNA extraction and GWAS genotyping. After the initial screening, five individuals were excluded due to missing or deteriorated DNA sample. Subsequently, during the Axiom quality control procedure, four individuals were excluded due to failed experiments. Finally, a total of 387 pairs (*n* = 775 individuals), with DNA sample, accurate genomic and clinical data were included in the GenCOLT biobank. **B** Steps for GWAS genotyping data cleaning. The Axiom PMRA chip used for GWAS genotyping covers 902,560 SNPs. According to the manufacturer guidelines, 852,344 SNPs passed the primary technological quality controls. Upstream SNP imputation, we excluded SNPs with high level of missingness (>2%), with low frequency (<1%) and not respecting the HWE (*p* < 10^-6^). Overall, GenCOLT contains 7.3 million high-quality SNP genotypes (DR or r^2^ > 0.8) for 387 LT pairs. *N.B. R, recipient; D, donor; GWAS, genome-wide association study; QC, quality control; SNP, single nucleotide polymorphism; MAF, minor allele frequency; HWE, Hardy-Weinberg equilibrium*.

To ensure the reliability and quality of the genomic data (Fig. 1A), we implemented several essential quality control (QC) steps. First, we performed the technological QC using AxAs (Axiom Analysis Suite) only to retain high-quality individuals, plates, and genetic variants. For individuals, a DishQC >0.82 ensured the proper separation of AT and GC fluorescence signals from noise in nonpolymorphic test probes, and the sample proportion with an assigned genotype (or call rate) was set to >97%. Similarly, we applied a call rate of >98.5% and >95% for plates and genetic variants, respectively. In addition, we evaluated for each SNP the genotype cluster quality assignment (Fisher’s linear discrimination >3.6) and the clear distinction between homozygous and heterozygous clusters (heterozygosity >95%). A total of 852,344 SNPs and 387 pairs (*n* = 775 individuals) passed this technological QC process.

We performed additional QC with PLINK [18] to prevent genotyping errors. We checked individuals with missingness >2% and evaluated the relatedness between samples; however, no individuals were excluded at this stage. For SNPs, we excluded those with missingness >2% and deviations from the Hardy-Weinberg equilibrium (*p* < 10^-6^, Fig. 1B). To address missing data, we employed imputation methods using the TOPMED [19] tool and TOPMED reference panel for SNP imputation. For *HLA* imputation, we employed HIBAG [20] along with a global reference panel (multiethnic samples from 1000 Genomes Project) [21, 22]. In both cases, we only retained SNPs and HLA alleles with high imputation quality (*r*^2^ > 0.8).

Finally, we carefully compared the imputed sex and *HLA* one-field alleles with sex and *HLA* allele information from clinical records to prevent potential errors of sample management during genotyping. As a result, GenCOLT collected robust high-quality imputed GWAS data for 7,337,433 SNPs and including 387 pairs (*n* = 775 individuals).

#### Genetic ancestry

To further describe GenCOLT, we assessed donors and recipients genetic ancestry using ADMIXTURE [23] and principal component analyses (PCA) from GWAS SNPs with a minor allele frequency (MAF) ≥ 1%. By comparing GenCOLT with the diverse 1000 Genomes Project [24] populations (*n* = 2,504 individuals from African (AFR), American (AMR), East Asian (EAS), European (EUR), and South Asian (SAS) reference populations), we aimed at detecting potential population stratification and at capturing ancestry-related variability in GenCOLT. ADMIXTURE defined genetic ancestry percentages per individual with a detailed breakdown within the five major ancestral groups (African, American, East Asian, European and South Asian). Each donor and recipient were attributed to an ancestry group when the ancestry percentage was ≥80%. When their ancestry did not meet the criterion of at least 80% contribution from any of the five major ancestral populations, the individuals were classified as admixed.

### Statistical analysis

Descriptive statistics were expressed as mean ± standard deviation and the min-max range for continuous variables, and as percentage for categorical variables. Difference among groups was tested using one-way ANOVA and chi-square tests for categorical variables. Kaplan-Meier analysis was performed to estimate the 5-year and 10-year survival after LT, and the differences in survival rate were compared using a log-rank test. All the analyses were performed in R 4.2.1. Two-sided *p*-values were considered significant for *p* < 0.001 to account for multiple testing, and nominally significant for *p* < 0.05.

## Results

### GenCOLT demographics and clinical characteristics

GenCOLT incorporates clinical data related to the donor (Table 1) and the recipient (Table 2). Women represented 43.9% donors and 44.9% recipients. The mean age was 48 years-old and the BMI was 21.8 (kg/m²) for recipients. The primary cause of death for donors was stroke with 53.6%. The main underlying disease leading to LT was chronic obstructive pulmonary disease (COPD) (44.6%), followed by cystic fibrosis (21.4%), interstitial lung disease (16.3%) and pulmonary hypertension (4.2%).

**Table 1:** Demographic table for GenCOLT donors.

Donors (*n* = 392)	
Sex (Female)	172 (43.9%)
Age in years	
Mean (SD)	45.37 (14.92)
Donor death type	
- Missing data	9 (2.3%)
- Anoxia	51 (13.0%)
- Other	17 (4.3%)
- Intoxication	1 (0.3%)
- Meningitis	4 (1.0%)
- Public road accident trauma	52 (13.3%)
- Non-public road accident trauma	46 (11.7%)
- Tumor	2 (0.5%)
- Stroke	210 (53.6%)

**Table 2:** Demographic table for GenCOLT recipients.

Recipients (*n* = 392)	
Sex (Female)	176 (44.9%)
Age (years)	
Mean (SD)	47.86 (14.18)
Body Mass Index in kg/ m²	
Mean (SD)	21.84 (4.38)
Underlying Disease	
- Cystic fibrosis	84 (21.4%)
- COPD / Emphysema	175 (44.6%)
- Pulmonary hypertension	16 (4.2%)
- Interstitial lung disease	64 (16.3%)
- Other	53 (13.5%)
Transplantation Procedure	
- Missing	2 (0.5%)
- Bilateral lung	335 (85.5%)
- Heart and lung	8 (2.0%)
- Single lung	47 (12.0%)
Cold Ischemia in mins	
Mean (SD)	369.66 (77.54)
Phenotype	
- Missing	13 (3.3%)
- Azithromycin reversible allograft dysfunction	11 (2.8%)
- Bronchiolitis obliterans syndrome	54 (13.8%)
- On wait	6 (1.5%)
- Undefined	11 (2.8%)
- Mixed	8 (2.0%)
- Other	78 (19.9%)
- Restrictive allograft syndrome	9 (2.3%)
- NON-CLAD with azithromycin	77 (19.6%)
- NON-CLAD	125 (31.9%)
Follow-up time in months	
Mean (SD)	67.1 (32.2)

We compared all clinical variables between the GenCOLT and COLT groups (Tables S1–S3). Importantly, only the patients’ distribution between clinical centers and the preservation fluid usage (Perfadex *vs*. Celsior) were statistically different between both groups (*p* < 0.001), directly reflecting the accessibility to DNA samples within centers and difference of preservation fluid used by center. We will consider these features as covariates in future GWAS analyses. Additional variables, such as immunosuppressive or induction treatment, corticosteroids therapy and lung reduction, were nominally significant (*p* < 0.05) but did not significantly differ between COLT and GenCOLT when accounting for multiple testing. Overall, the GenCOLT recipients do not differ from the rest of the COLT cohort in terms of clinical data, pre, per and post transplantation. Additionally, we examined the four major underlying causes of LT, as different etiologies could differentially impact FEV1 spirometry values, and no significant difference was observed (Tables S4–S7).

### Survival analysis

**Fig. 2:**
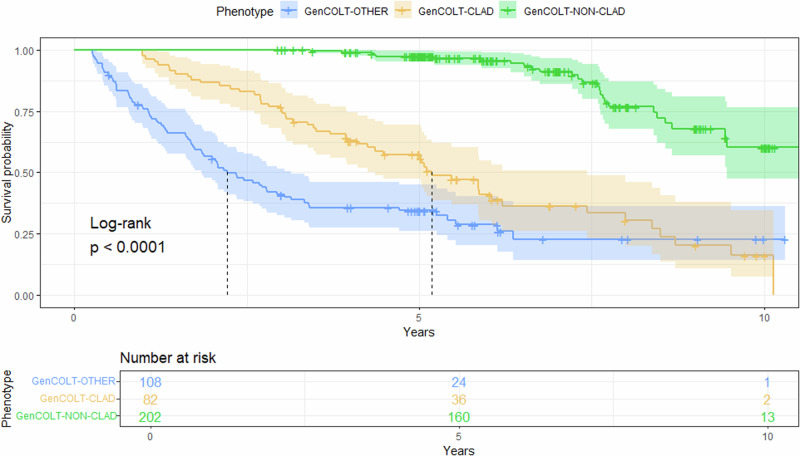
Kaplan–Meier lung graft survival curves according to the rejection phenotypes vs. the non-CLAD grafts in GenCOLT. The ‘CLAD’ subgroup includes the 4 ISHLT consensus phenotypes: BOS, RAS, mixed, and undefined. The ‘other’ subgroup refers to other types of primary rejection, including infectious-induced rejection and azithromycin-responsive allograft dysfunction (ARAD) in the first month’s post-surgery. *N.B. BOS, bronchiolitis obliterans syndrome; RAS, restrictive allograft syndrome; ARAD, Azithromycin reversible allograft dysfunction; ISHLT, International Society for Heart and Lung Transplantation*.

We described the post-transplantation survival in GenCOLT by stratifying according to clinical phenotypes: non-CLAD, CLAD, and other (Fig. 2). The median survival for CLAD and the other group was 62 and 26 months, respectively. For non-CLAD recipients, the survival rate was 97.4% at 5 years, and it declined to 60.2% at 10 years. For CLAD recipients, the survival rate was 57.3% at 5 years and it fell to 16.2% at 10 years. Finally, for the other group the survival rate was 34.4% at 5 years and it dropped to 22.8% at 10 years.

**Fig. 3:**
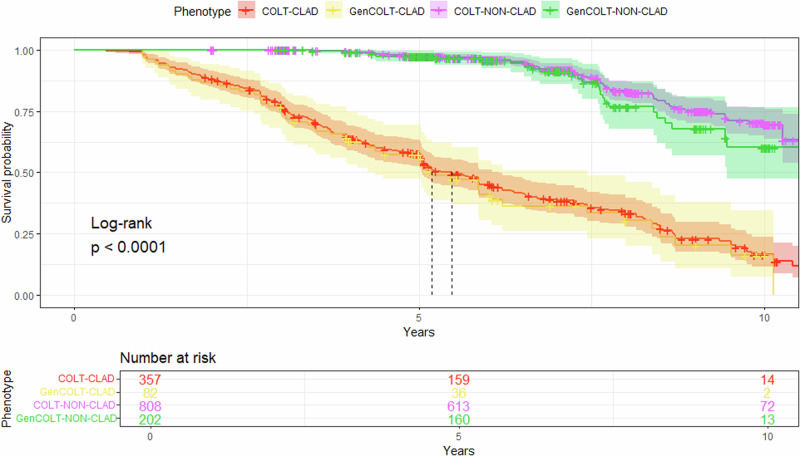
Kaplan–Meier lung graft curves according to the CLAD vs. non-CLAD phenotypes for COLT and GenCOLT. The ‘CLAD’ subgroup includes the 4 ISHLT consensus phenotypes: BOS, RAS, mixed, and undefined. *N.B. BOS, bronchiolitis obliterans syndrome; RAS, restrictive allograft syndrome*.

In a subsequent analysis, we compared the survival rates between COLT and GenCOLT patients (Fig. 3). The median survival for CLAD transplant patients from COLT and GenCOLT was 66 and 62 months, respectively. For non-CLAD COLT patients, the survival rate was 97.5% at 5 years and it declined to 70.5% at 10 years. For COLT patients with CLAD, the survival rate was 57.4% at 5 years and it dropped to 16.5% at 10 years. In both cohorts, the survival was significantly shorter for CLAD patients than for non-CLAD patients (log-rank *p* < 0.0001). There was no difference in lung allograft survival between COLT and GenCOLT for non-CLAD (*p* = 0.3) and CLAD (*p* = 0.6) patients. Overall, our analysis demonstrated similar survival rates for transplanted patients from COLT and GenCOLT.

### Data cleaning for GWAS

#### Robustness and quality of GenCOLT genetic data

We assessed the genotyping robustness and quality of our genetic data throughout the whole GenCOLT building process (DNA extraction, sample handling, normalization and genotyping, Fig. 1). Importantly, we compared genetically inferred sex and *HLA* alleles with information from medical records and we did not identify any discrepancy, confirming the proper sample management.

After careful standard quality controls for SNPs, we imputed 7,337,433 SNPs with accurate genotypes (r² > 0.8) from 400 K genotyped SNPs (Fig. 1B and S1). Before imputation, we observed an enrichment in rare variants in accordance with the PMRA design. After imputation, the number of SNPs with MAF > 2% has significantly increased across the whole MAF spectrum. All these high-quality genetic variants are available for subsequent association testing.

We compared *HLA* allele information available before and after imputation (Figure S2). We observed a considerable gain of allele calling for the *HLA-C* gene (from 1.1% before imputation to 100% after imputation). Importantly, *HLA* imputation preserved the distribution of allelic diversity while increasing the available *HLA* information. As expected, the *HLA-B* locus exhibits a higher level of polymorphism [20, 25, 26] compared to other *HLA* genes such as *HLA*-*DQB1* for example.

#### Genetic ancestry

**Fig. 4:**
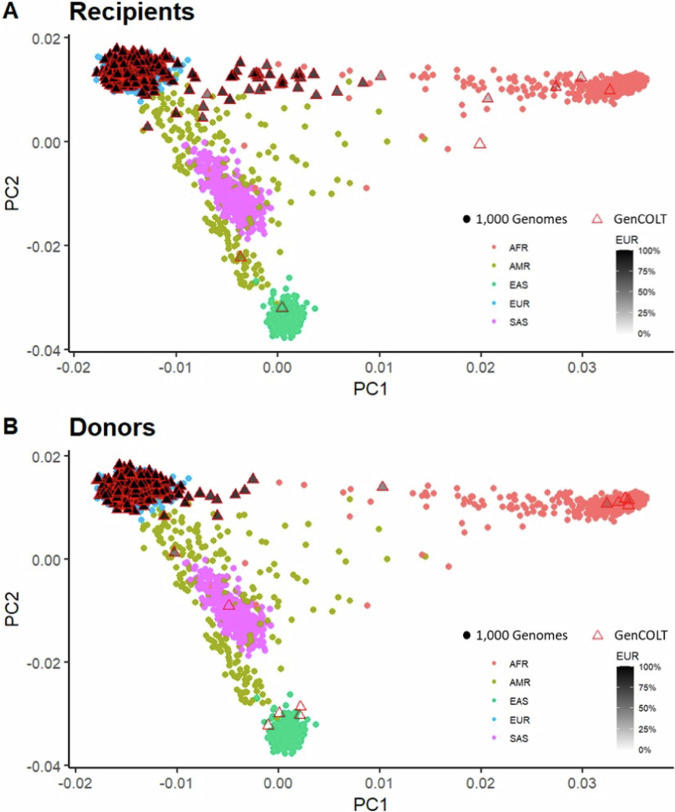
Projection of GenCOLT individuals alongside the 1000 Genomes Project reference individuals. **A** GenCOLT recipients and **B** GenCOLT donors. Using the GWAS SNP data, we projected GenCOLT individuals (represented by triangle) with the 1000 Genomes Project individuals (represented by circles) from five large reference populations. The triangle color refers to the percent of European ancestry for GenCOLT individuals as defined with ADMIXTURE (with darker shades indicating higher European ancestry). PCA principal component analysis, AFR African, AMR American, EAS East Asian, EUR European, SAS South Asian.

We conducted a PCA to examine genetic ancestry and population stratification within our genetic dataset in comparison with the reference populations from the 1000 Genomes Project [27]. This analysis revealed a distinct separation of individuals from the 1,000 Genomes Project into five clusters (Fig. 4) representing the five major reference ancestral populations. The GenCOLT samples were spread across all the reference populations with an important concentration among individuals of European ancestry. To go further, we defined genetic ancestry percentages within the five major ancestral groups per individual. When focusing on donor-recipient pairs, our analysis revealed that the majority of pairs (90.8%) shared European ancestry (Fig. S3). In addition, 7.6% pairs were composed of a European donor and an admixed recipient, and four pairs (0.5%) were composed of a European recipient and an East Asian donor. GenCOLT also gathered three pairs with at least one individual (donor or recipient) with admixed ancestry and five pairs with Asian ancestry. The remaining 2% pairs included donors and recipients of non-European ancestry.

## Discussion

The GenCOLT biobank represents a unique and valuable resource in Europe for investigating genetic and immunogenetic factors in LT. It was established as a COLT subpart, which has been a comprehensive source of clinical data on LT since 2009. Over a span of 10 years, we have created a robust and consistent multicentric cohort that accurately reflects the clinical practices and medical records of LT in France and Belgium. Of the hundreds of available clinical variables, only two are significantly different between GenCOLT and COLT (center and preserving liquid), which is why we consider GenCOLT to be a smaller but accurate representation of COLT, and therefore a snapshot of LT clinical practice between 2009 and 2018. GenCOLT hence offers a comprehensive resource for studying the genetic determinants of post-LT outcomes and complications, including CLAD, ARAD, and a wide variety of other phenotypes such as bacterial, viral and fungal infections, which are common and can significantly impact morbidity and mortality (all the phenotypes available in GenCOLT are summarized in Tables S1–3).

To ensure data homogeneity and quality, the GenCOLT genomic data were generated using a standardized experimental pipeline, including the same genotyping chips, technological protocols and analytical pipelines. Rigorous quality controls were implemented to guarantee the highest data quality. Additional checks were conducted to verify sex and *HLA* matching between genetically inferred information and medical records, minimizing the risk of sample mishandling. This was particularly important considering the retrieval of samples that were stored for more than 10 years, which increased the potential for human errors.

Furthermore, SNP to *HLA* allele imputation was also performed to enhance the immunogenetic genotypes within the GenCOLT dataset. This imputation process addressed the issue of incomplete *HLA* data in the COLT clinical records, particularly for *HLA-C*, where 98.9% data was missing in both recipients and donors. By retrospectively filling these missing gaps, SNP to *HLA* imputation provided consistent *HLA* annotations for future immunogenetic association testing.

Additionally, by projecting SNP genetic data from GenCOLT and individuals of the 1000 Genomes Project, we were able to assess the genetic ancestry of donors and recipients without directly collecting ancestry background information, as it is prohibited in France and Belgium. This approach helped prevent population stratification and provided insights into the genetic ancestry within the GenCOLT dataset. Overall, the establishment of GenCOLT involved meticulous processes to ensure data conformity, sample traceability, and comprehensive genetic and immunogenetic information. It represents a valuable resource for studying LT outcomes and its associated molecular factors in a standardized and controlled manner.

However, the GenCOLT cohort has some limits. First of all, the sample size is limited compared with studies on other solid organ transplantation such as kidney transplantation [28], which might limit our statistical power for discovery in our future GWAS analyses. Similarly, GenCOLT is essentially composed of European individuals, and as such, we will only be able to draw conclusions on the genetic risk factors for lung graft rejection at the European level. To address this limitation, we are planning future collaborations with partners from the International Genetics and Translational Research in Transplantation Network (iGeneTRAiN) [29, 30], an international collaborative effort and the largest genetic initiative focusing on better understanding transplant rejection and complications to date. Meta-analyses and additional patient recruitment will increase the statistical power for discoveries, while potentially increasing the genetic diversity of the study population.

In conclusion, GenCOLT stands as one of the most extensive LT DNA cohorts worldwide, encompassing 387 donor-recipient pairs (*n* = 775 individuals). This comprehensive cohort not only comprises clinical data but also genetic information for both donors and recipients, making it an invaluable resource for investigating the underlying mechanisms of CLAD, as well as other LT outcomes and complications. The genetic profiles of the recipients are likely to significantly impact a wide range of transplantation outcomes, from their susceptibility to experiencing rejection, response to medications (pharmacogenetics), to the development of various medical conditions linked to the prolonged use of immunosuppressive treatments [31]. Furthermore, the genetic makeup of the organ donors may also serve as a predictor for the function of the transplanted organ. Finally, the genetic compatibilities between the donor and recipient could play a crucial role in influencing both the likelihood of rejection and the long-term survival of transplanted organs. The identification of *HLA* and non-*HLA* genetic associations within GenCOLT would significantly enhance our understanding of CLAD and other LT outcomes, and may uncover potential therapeutic targets. Moreover, the identification of predictive biomarkers in GenCOLT would help to stratify individual risk at an early stage, before the onset of symptoms, ultimately improving personalized and predictive medicine in the field of lung transplantation. The wealth of information within GenCOLT therefore opens up new avenues for research and has the potential to advance our knowledge and management of lung transplant patients.

## Supplementary information

Supplementary Material

## Data Availability

The GenCOLT data are available from our CR2TI team. Data and summary statistics are available upon request following approval from the GenCOLT steering committee and ethics committee to ensure data protection and privacy in compliance with French and European laws. Collaborations are encouraged through specific research projects using GenCOLT data or through enriching the existing cohort with new patients. Potential collaborators are invited to contact the primary investigator Sophie Limou: sophie.limou@univ-nantes.fr.
