# Supplementary material for: Description and first insights on a large genomic biobank of lung transplantation
Source: Eur J Hum Genet. 2024 Aug 20;33(3):304–11. doi: 10.1038/s41431-024-01683-y (PMC11893754; doi:10.1038/s41431-024-01683-y)
Supplement: Supplementary file 1 — Supplementary Material [file 41431_2024_1683_MOESM1_ESM.docx]

Supplementary material for

Description and first insights on a large genomic biobank of lung transplantation (Brocard et al.)

Table of contents

[**Supplementary information: COLT consortium** 3](#_Toc173334417)

[**Supplementary material and methods:** 6](#_Toc173334418)

[**Supplementary Table 1:** Characteristics of the donors in GenCOLT and the rest of the COLT cohort 7](#_Toc173334419)

[**Supplementary Table 2**: Characteristics of the recipients in GenCOLT and the rest of the COLT cohort 9](#_Toc173334420)

[**Supplementary Table 3**: Characteristics of the transplanted patients’ in GenCOLT and the rest of the COLT cohort 11](#_Toc173334421)

[**Supplementary Table 4**: Functional characteristics in GenCOLT and the rest of the COLT cohort for chronic obstructive pulmonary disease recipients. 16](#_Toc173334422)

[**Supplementary Table 5**: Functional characteristics in GenCOLT and the rest of the COLT cohort for cystic fibrosis recipients 17](#_Toc173334423)

[**Supplementary Table 6**: Functional characteristics in GenCOLT and the rest of the COLT cohort in interstitial lung disease recipients 18](#_Toc173334424)

[**Supplementary Table 7**: Functional characteristics in GenCOLT and the rest of the COLT cohort in pulmonary hypertension recipients 20](#_Toc173334425)

[**Supplementary Figure 1**: SNP enrichment through the SNP imputation process. 22](#_Toc173334426)

[**Supplementary Figure 2**: Distribution of *HLA* alleles in GenCOLT individuals according to typed vs. imputed *HLA-A*, *HLA-B*, *HLA-C*, *HLA*-*DRB1* and *HLA*-*DQB1* gene alleles. 23](#_Toc173334427)

[**Supplementary Figure 3**: Genetic ancestry matching between donors and recipients from GenCOLT pairs. 24](#_Toc173334428)

## **Supplementary information: COLT consortium**

Elodie Blanchard^8^, Xavier Demant^8^, Virginie Hulo^8^, Maria Ruiz-Patino^13^, Maarten Vander Kuylen^13^, Youri Sokolow^13^, Constantin Stefanidis^13^, Isabelle Huybrechts^13^, Laurent Perrin^13^, Fabio Taccone^13^, Isabelle Etienne^13^, Christiane Knoop^13^, Anna Roussoulières^13^, Maya Hites^13^, Agnes Lambert^13^, Axelle Hemelsoet^13^, Pierrick Bedouch^9^, Amandine Briault^9^, Loic Falque^9^, Quentin Perrier^9^, Christel Saint Raymond^9^, Samarmar Chacaroun^9^, Yoann Gioria^9^, Joane Quentin^9^, Renaud Grima^14^, Gabrielle Drevet^14^, Jean-Michel Maury^14^, François Tronc^14^, Philippe Portan^14^, Jean-François Mornex^14^, Claire Merveilleux Du Vignaud^14^, Eva Chatron^14^, Jean Charles Glérant^14^, Ségolène Turquier^14^, Salim Si Mohamed^14^, Vincent Cottin^14^, Lara Chalabresse^14^, Chantal Dubois^14^, Aurélie Rea^14^, Médéric Reignier^14^, Julia Canterini^14^, Nicolas Carlier^16^, Véronique Boussaud^16^, Romain Guillemain^16^, Xavier-Benoit D’Journo^7^, Pascale-Alexandre Thomas^7^, Delphine Trousse^7^, Geoffrey Brioude^7^, David Boulate^7^, Alex Fourdrain^7^, Fabienne Bregeon^7^, Stéphane Delliaux^7^, Martine Reynaud-Gaubert^7^, Bérengère Coltey^7^, Nadine Dufeu^7^, Benjamin Coiffard^7^, Julien Bermudez^7^, Ana Nieves^7^, Hervé Dutau^7^, Julie Tronchetti^7^, Jean-Yves Gaubert^7^, Paul Habert^7^, Mathieu Di Biscéglie^7^, Agnes Basire^7^, Pascal Pedini^7^, Florence Daviet^7^, Christophe Guervilly^7^, Sami Hraiech^7^, Jean Marie Forel^7^, Louis Delamarre^7^, Aude Charvet^7^, Ines Gragueb-Chatti^7^, Pierre Mora^7^, Daniel Laurent^7^, Sophie Giusiano^7^, Jean-Philippe Dales^7^, Mélanie Gaubert^7^, Marc Laine^7^, Philippe Lacoste^1^, Christian Perigaud^1^, Jean-Christian Roussel^1^, Thomas Senage^1^, Antoine Mugniot^1^, Isabelle Danner^1^, Adrien Tissot^1^, Charlotte Bry^1^, Morgane Penhouet^1^, Emmanuelle Eschapasse^1^, Delphine Horeau-Langlard^1^, François-Xavier Blanc^1^, Thierry Lepoivre^1^, Mickael Vourch^1^, Sophie Brouard^1^, Richard Danger^1^, Megguy Bernard^1^, Elodie Godard^1^, Régine Valéro^1^, Karine Maugendre^1^, Eugénie Durand^1^, Nataliya Yeremenko^1^, Aurore Foureau^1^, David Lair^1^, Géraldine Gallot^1^, Mathilde Berthome^1^, Jérôme Le Pavec^10^, Gaëlle Dauriat^10^, Pauline Pradere^10^, Séverine Feuillet^10^, Samuel Dolidon^10^, Chahine Medraoui^10^, Pierre Gazengel^10^, Adrian Crutu^10^, Amir Hanna^10^, Elie Fabre^10^, Olaf Mercier^10^, Delphine Mitilian^10^, Justin Issard^10^, Dominique Fabre^10^, Yves Castier^15^, Pierre Mordant^15^, Pierre Cerceau^15^, Antoine Girault^15^, Arnaud Roussel^15^, Enora Atchade-Thierry^15^, Sylvain Jean-Baptiste^15^, Sandrine Boudinet^15^, Sébastien Tanaka^15^, Aurélie Gouel^15^, Philippe Montravers^15^, Nathalie Zappella^15^, Aurélie Snauwaert^15^, Parvine Tashk^15^, Brice Lortat-Jacob^15^, Tiphaine Goletto^15^, Domitille Mouren^15^, Lise Morer^15^, Mathilde Salpin^15^, Hervé Mal^15^, Armelle Marceau^15^, Gaëlle Weisenburger^15^, Vincent Bunel^15^, Adèle Sandot^15^, Kinan El Husseini^15^, Pierre Halitim^15^, Lucie Genet^15^, Sabrina Trigueiros^15^, Alice Savary^15^, Hakima Rabia^15^, Pierre-Emmanuel Falcoz^6^, Anne Olland^6^, Charlotte Ponte^6^, Charles Tacquard^6^, Garib Ajob^6^, Olivier Collange^6^, Antoine Pons^6^, Xavier Delabranche^5^, Olivier Helms^6^, Anne Roche^6^, Benjamin Renaud-Picard^6^, Romain Kessler^6^, Tristan Degot^6^, Sandrine Hirschi^6^, Armelle Schuller^6^,Anne Dory^6^,Florence Toti^6^,Nadia Benkirane-Jessel^6^, Laurence Kessler^6^,Julien Stauder^6^, Edouard Sage^11^, Francois Parquin^11^, Sandra De Miranda^11^, Clément Picard^11^, Antoine Roux^11^, Olivier Brugière^11^, Béatrice D’Urso^11^, Marc Stern^11^, Akounach Mbarka^11^, Antoine Magnan^17^, Quentin Marquant^11^, Isabelle Schwartz^11^, Helene Salvator^11^, Tiffany Pascreau^11^, Thomas Villeneuve^12^, Marion Dupuis^12^, Marlène Murris-Espin^12^, Pierre Rabinel^12^, Laurent Brouchet^12^, Laure Crognier^12^, Olivier Mathe^12^, Frédérique Legenne^12^, Myriam Barthes^12^, Blandine Vilquin^12^, Anne-Laure Costes^12^, Isabelle Recoche^12^, Anne Bergeron^18^, Gregory Berra^18^ and Angela Koutsokera^18^.

^1^ Nantes Université, CHU Nantes, Centrale Nantes, Inserm, Center for Research in Transplantation and Translational Immunology, UMR 1064, ITUN, Nantes, France

^6^ Department of Respiratory Medicine and Strasbourg Lung Transplant Program, Hôpitaux Universitaires de Strasbourg, Strasbourg, France; Université de Strasbourg, Inserm UMR 1260, Strasbourg, France

^7^ Aix Marseille Univ, Department of Respiratory Medicine and Lung Transplantation, APHM, Hôpital Nord, Marseille, France

^8^ Service de Pneumologie, Centre Hospitalier Universitaire de Bordeaux, Pessac, France

^9^ Service Hospitalier Universitaire de Pneumologie et Physiologie, CHU Grenoble Alpes, Pôle Thorax et Vaisseaux, Grenoble, France

^10^ Service de Pneumologie et Transplantation Pulmonaire, Groupe hospitalier Marie-Lannelongue -Saint Joseph, Le Plessis-Robinson, Université Paris-Saclay, Le Kremlin Bicêtre, UMR_S 999, Université Paris–Sud, INSERM France

^11^ Pneumology, Adult Cystic Fibrosis Center and Lung Transplantation Department Hôpital Foch, Suresnes, Université de Versailles Saint Quentin Paris-Saclay, INRAe UMR 0892, Paris Transplant Group, Paris, France

^12^ CHU Toulouse, Service de Pneumologie, Université Toulouse III-Paul Sabatier, Toulouse, France

^13^ Service de Pneumologie, CHU Erasme, Bruxelles, Belgium

^14^ Université de Lyon, Université Lyon 1, PSL, EPHE, INRAE, IVPC, hospices civils de Lyon, groupement hospitalier est, service de pneumologie, Orphalung, RESPIFIL Lyon, France

^15^ APHP Nord-Université Paris Cité, Hôpital Bichat, Service de Pneumologie B et Transplantation Pulmonaire, Université Paris Cité, PHERE UMRS 1152, Paris, France

^16^ APHP, Service de Pneumologie, Hôpital Cochin, Paris, France

^17^ Hôpital Foch, Université de Versailles Saint Quentin Paris-Saclay, INRAe UMR 0892, France

^18^ Service de Pneumologie, Hôpitaux Universitaires de Genève, Rue Gabrielle-Perret-Gentil 4, 1211 Genève 14

## **Supplementary material and methods:**

The clinical records and follow-up data from each participating center are centralized in a secured online database coordinated by the Nantes University Hospital. This database contains a comprehensive set of over 300 variables encompassing various aspects of the lung transplantation process, including demographic information about the recipient, the donor, the transplantation procedure, and subsequent recipient’s follow-up. In the present work, we only present the 67 variables that are mostly used in clinical practice. As per the routine COLT protocol, follow-up visits were conducted at 1-month and 6-month after transplantation, and then every 6 months for a period of up to 5 years for biological sample (blood) collection, and up to 10 years for clinical data. The prospective nature of both the database and the biobank allows the identification of predisposing clinical and biological markers in patients who developed CLAD during follow-up.

## **Supplementary Table 1:** Characteristics of the donors in GenCOLT and the rest of the COLT cohort

|  | COLT (N=1413) | GenCOLT (N=392) | p value |
| --- | --- | --- | --- |
| **Sex** |  |  | 0.909 (1) |
| - Missing | 54 | 4 |  |
| - Men | 761 (56.0%) | 216 (55.7%) |  |
| - Women | 598 (44.0%) | 172 (44.3%) |  |
| **Age (years)** |  |  | 0.807 (2) |
| - Missing | 51 | 5 |  |
| - Mean (SD) | 45.16 (15.31) | 45.370 (14.92) |  |
| - Range | 9.00 - 73.00 | 18.00 - 71.00 |  |
| **Days in reanimation** |  |  | 0.343 (2) |
| - Missing | 91 | 20 |  |
| - Mean (SD) | 2.76 (2.84) | 2.60 (2.49) |  |
| - Range | 0.00 - 39.00 | 0.00 - 19.00 |  |
| **Cause of death** |  |  | 0.926 (1) |
| - Missing | 72 | 9 |  |
| - Anoxia | 174 (13.0%) | 51 (13.3%) |  |
| - Intoxication | 7 (0.5%) | 1 (0.3%) |  |
| - Meningitis | 14 (1.0%) | 4 (1.0%) |  |
| - Non-PRA Trauma | 192 (14.3%) | 46 (12.0%) |  |
| - Other | 47 (3.5%) | 17 (4.4%) |  |
| - PRA Trauma | 181 (13.5%) | 52 (13.6%) |  |
| - Tumor | 9 (0.7%) | 2 (0.5%) |  |
| - Stroke | 717 (53.5%) | 210 (54.8%) |  |
| **Blood type** |  |  | 0.694 (1) |
| - Missing | 57 | 5 |  |
| - A | 546 (40.3%) | 146 (37.7%) |  |
| - AB | 27 (2.0%) | 9 (2.3%) |  |
| - B | 127 (9.4%) | 33 (8.5%) |  |
| - O | 656 (48.4%) | 199 (51.4%) |  |
| **Tobacco** |  |  | 0.918 (1) |
| - Missing | 108 | 13 |  |
| - No | 795 (60.9%) | 232 (61.2%) |  |
| - Yes | 510 (39.1%) | 147 (38.8%) |  |
| **Number of packages per year** |  |  | 0.099 (2) |
| - Missing | 1316 | 373 |  |
| - Mean (SD) | 18.34 (14.15) | 24.32 (15.13) |  |
| - Range | 1.00 - 70.00 | 1.00 - 60.00 |  |
| **Type of sampling** |  |  | 0.935 (1) |
| - Missing | 1271 | 366 |  |
| - Brain death | 137 (96.5%) | 25 (96.2%) |  |
| - Maastricht III | 5 (3.5%) | 1 (3.8%) |  |
| **HIV antibodies** |  |  | 0.295 (1) |
| - Missing | 71 | 10 |  |
| - Negative | 1337 (99.6%) | 379 (99.2%) |  |
| - Positive | 5 (0.4%) | 3 (0.8%) |  |
| **Toxoplasmosis antibodies** |  |  | 0.661 (1) |
| - Missing | 152 | 30 |  |
| - Negative | 489 (38.8%) | 145 (40.1%) |  |
| - Positive | 772 (61.2%) | 217 (59.9%) |  |
| **EBV antibodies** |  |  | 0.476 (1) |
| - Missing | 63 | 11 |  |
| - Negative | 92 (6.8%) | 30 (7.9%) |  |
| - Positive | 1258 (93.2%) | 351 (92.1%) |  |
| **HBC antibodies** |  |  | 0.950 (1) |
| - Missing | 272 | 88 |  |
| - Negative | 1108 (97.1%) | 295 (97.0%) |  |
| - Positive | 33 (2.9%) | 9 (3.0%) |  |
| **CMV antibodies** |  |  | 0.395 (1) |
| - Missing | 52 | 3 |  |
| - Negative | 698 (51.3%) | 190 (48.8%) |  |
| - Positive | 663 (48.7%) | 199 (51.2%) |  |
| **P/F ratio** |  |  | 0.018 (2)(4) |
| - Missing | 311 | 83 |  |
| - Mean (SD) | 358.92 (151.80) | 381.36 (131.86) |  |
| - Range | 1.00 - 629.00 | 2.00 - 605.00 |  |

1. Pearson’s Chi-squared test
2. Linear Model ANOVA
3. Significant
4. Nominally significant

*N.B. HIV, human immunodeficiency virus; EBV, Epstein-Barr virus; HBC, Hepatitis B; CMV, Cytomegalovirus; P/F ratio, ratio of arterial oxygen partial pressure (PaO2 in mmHg) to fractional inspired oxygen (FiO2); PRA, public road* *accident; percentages excludes missing data.*

## **Supplementary Table 2**: Characteristics of the recipients in GenCOLT and the rest of the COLT cohort

|  | COLT (N=1413) | GenCOLT (N=392) | p value |
| --- | --- | --- | --- |
| **Sex** |  |  | 0.418 (1) |
| - Men | 746 (52.8%) | 216 (55.1%) |  |
| - Women | 667 (47.2%) | 176 (44.9%) |  |
| **Height (cm)** |  |  | 0.236 (2) |
| - Missing | 45 | 6 |  |
| - Mean (SD) | 166.78 (9.23) | 167.41 (8.98) |  |
| - Range | 130.00 - 190.00 | 146.00 - 190.00 |  |
| **Age (years)** |  |  | 0.013 (2)(4) |
| - Missing | 41 | 1 |  |
| - Mean (SD) | 45.77 (14.74) | 47.857 (14.177) |  |
| - Range | 12.00 - 69.00 | 18.00 - 69.00 |  |
| **Weight (kg)** |  |  | 0.121 (2) |
| - Missing | 49 | 7 |  |
| - Mean (SD) | 60.34 (15.06) | 61.69 (15.11) |  |
| - Range | 24.00 - 117.00 | 36.00 - 110.00 |  |
| **Body mass index (kg/m2)** |  |  | 0.240 (2) |
| - Missing | 53 | 8 |  |
| - Mean (SD) | 21.54 (4.42) | 21.84 (4.38) |  |
| - Range | 13.46 - 40.01 | 13.55 - 34.78 |  |
| **Ischemic cardiopathy** |  |  | 0.294 (1) |
| - Missing | 1086 | 320 |  |
| - No | 255 (78.0%) | 52 (72.2%) |  |
| - Yes | 72 (22.0%) | 20 (27.8%) |  |
| **High blood pressure** |  |  | 0.166 (1) |
| - Missing | 1001 | 295 |  |
| - No | 240 (58.3%) | 49 (50.5%) |  |
| - Yes | 172 (41.7%) | 48 (49.5%) |  |
| **Diabetes** |  |  | 0.759 (1) |
| - Missing | 952 | 290 |  |
| - No | 202 (43.8%) | 43 (42.2%) |  |
| - Yes | 259 (56.2%) | 59 (57.8%) |  |
| **Cardiac insufficiency** |  |  | 0.661 (1) |
| - Missing | 1123 | 336 |  |
| - No | 269 (92.8%) | 51 (91.1%) |  |
| - Yes | 21 (7.2%) | 5 (8.9%) |  |
| **Gastroesophageal reflux** |  |  | 0.614 (1) |
| - Missing | 1024 | 309 |  |
| - No | 218 (56.0%) | 44 (53.0%) |  |
| - Yes | 171 (44.0%) | 39 (47.0%) |  |
| **Hepatic damage** |  |  | 0.353 (1) |
| - Missing | 1119 | 333 |  |
| - No | 253 (86.1%) | 48 (81.4%) |  |
| - Yes | 41 (13.9%) | 11 (18.6%) |  |
| **Dyslipidemia** |  |  | 0.015 (1)(4) |
| - Missing | 1063 | 314 |  |
| - No | 255 (72.9%) | 46 (59.0%) |  |
| - Yes | 95 (27.1%) | 32 (41.0%) |  |
| **Renal insufficiency** |  |  | 0.490 (1) |
| - Missing | 1133 | 338 |  |
| - No | 270 (96.4%) | 51 (94.4%) |  |
| - Yes | 10 (3.6%) | 3 (5.6%) |  |
| **Pulmonary embolism** |  |  | 0.548 (1) |
| - Missing | 1083 | 328 |  |
| - No | 257 (77.9%) | 52 (81.2%) |  |
| - Yes | 73 (22.1%) | 12 (18.8%) |  |
| **Tuberculosis** |  |  | 0.174 (1) |
| - Missing | 597 | 140 |  |
| - No | 804 (98.5%) | 251 (99.6%) |  |
| - Yes | 12 (1.5%) | 1 (0.4%) |  |
| **Hemoptysis** |  |  | 0.582 (1) |
| - Missing | 1086 | 321 |  |
| - No | 254 (77.7%) | 53 (74.6%) |  |
| - Yes | 73 (22.3%) | 18 (25.4%) |  |
| **Dialysis** |  |  | 0.336 (1) |
| - Missing | 107 | 19 |  |
| - No | 1214 (93.0%) | 352 (94.4%) |  |
| - Yes | 92 (7.0%) | 21 (5.6%) |  |
| **Non invasive ventilation** |  |  | 0.256 (1) |
| - Missing | 83 | 24 |  |
| - No | 762 (57.3%) | 223 (60.6%) |  |
| - Yes | 568 (42.7%) | 145 (39.4%) |  |
| **Oxygenotherapy** |  |  | 0.220 (1) |
| - Missing | 49 | 6 |  |
| - No | 143 (10.5%) | 49 (12.7%) |  |
| - Yes | 1221 (89.5%) | 337 (87.3%) |  |
| **CMV antibodies** |  |  | 0.261 (1) |
| - Missing | 21 | 2 |  |
| - Negative | 673 (48.3%) | 176 (45.1%) |  |
| - Positive | 719 (51.7%) | 214 (54.9%) |  |
| **EBV antibodies** |  |  | 0.980 (1) |
| - Missing | 25 | 3 |  |
| - Negative | 104 (7.5%) | 29 (7.5%) |  |
| - Positive | 1284 (92.5%) | 360 (92.5%) |  |
| **Toxoplasmosis antibodies** |  |  | 0.914 (1) |
| - Missing | 80 | 27 |  |
| - Negative | 552 (41.4%) | 150 (41.1%) |  |
| - Positive | 781 (58.6%) | 215 (58.9%) |  |
| **HBC antibodies** |  |  | 0.666 (1) |
| - Missing | 35 | 4 |  |
| - Negative | 1341 (97.3%) | 376 (96.9%) |  |
| - Positive | 37 (2.7%) | 12 (3.1%) |  |
| **Blood type** |  |  | 0.487 (1) |
| - Missing | 195 | 57 |  |
| - A | 518 (42.5%) | 138 (41.2%) |  |
| - AB | 42 (3.4%) | 9 (2.7%) |  |
| - B | 136 (11.2%) | 31 (9.3%) |  |
| - O | 522 (42.9%) | 157 (46.9%) |  |

1. Pearson’s Chi-squared test
2. Linear Model ANOVA
3. Significant
4. Nominally significant

*N.B. CMV, Cytomégalovirus ; EBV, Epstein-Barr virus ; percentages excludes missing data.*

## **Supplementary Table 3**: Characteristics of the transplanted patients’ in GenCOLT and the rest of the COLT cohort

|  | COLT (N=1413) | GenCOLT (N=392) | p value |
| --- | --- | --- | --- |
| **Phenotype** |  |  | 0.954 (1) |
| - Missing | 215 | 13 |  |
| - Azithromycin-responsive allograft dysfunction (ARAD) | 29 (2.4%) | 11 (2.9%) |  |
| - Bronchiolitis obliterans syndrome (BOS) | 187 (15.6%) | 54 (14.2%) |  |
| - Undefined | 32 (2.7%) | 11 (2.9%) |  |
| - Mixte | 21 (1.8%) | 8 (2.1%) |  |
| - On wait | 26 (2.2%) | 6 (1.6%) |  |
| - Other | 262 (21.9%) | 78 (20.6%) |  |
| - Restrictive allograft syndrome (RAS) | 35 (2.9%) | 9 (2.4%) |  |
| - Non-CLAD | 361 (30.1%) | 125 (33.0%) |  |
| - Non-CLAD with azithromycin | 245 (20.5%) | 77 (20.3%) |  |
| **Center** |  |  | **< 0.001** (1)(3) |
| - Bordeaux | 177 (12.5%) | 46 (11.7%) |  |
| - Bruxelles | 21 (1.5%) | 14 (3.6%) |  |
| - Grenoble | 58 (4.1%) | 35 (8.9%) |  |
| - Le-Plessis-Robinson | 141 (10.0%) | 28 (7.1%) |  |
| - Lyon | 106 (7.5%) | 13 (3.3%) |  |
| - Marseille | 216 (15.3%) | 81 (20.7%) |  |
| - Nantes | 80 (5.7%) | 23 (5.9%) |  |
| - Paris-Bichat | 56 (4.0%) | 9 (2.3%) |  |
| - Paris-HEGP | 45 (3.2%) | 5 (1.3%) |  |
| - Strasbourg | 180 (12.7%) | 89 (22.7%) |  |
| - Suresnes | 275 (19.5%) | 25 (6.4%) |  |
| - Toulouse | 58 (4.1%) | 24 (6.1%) |  |
| **Long term corticosteroid therapy** |  |  | 0.034 (1)(4) |
| - Missing | 907 | 283 |  |
| - No | 105 (20.8%) | 13 (11.9%) |  |
| - Yes | 401 (79.2%) | 96 (88.1%) |  |
| **Immunosuppresive treatment** |  |  | 0.045 (1)(4) |
| - Missing | 1116 | 339 |  |
| - No | 189 (63.6%) | 26 (49.1%) |  |
| - Yes | 108 (36.4%) | 27 (50.9%) |  |
| **Immunosuppresive treatment type** |  |  | 0.831 (1) |
| - Missing | 1341 | 371 |  |
| - Azathioprine | 8 (11.1%) | 1 (4.8%) |  |
| - Cyclosporine | 4 (5.6%) | 2 (9.5%) |  |
| - Mycophenolate mofétil | 6 (8.3%) | 1 (4.8%) |  |
| - Other | 50 (69.4%) | 16 (76.2%) |  |
| - Tacrolimus | 4 (5.6%) | 1 (4.8%) |  |
| **Forced expiratory volume in one second at transplant (FEV1)** |  |  | 0.835 (2) |
| - Missing | 65 | 14 |  |
| - Mean (SD) | 31.77 (19.50) | 31.532 (19.60) |  |
| - Range | 3.35 - 122.00 | 10.00 - 110.40 |  |
| **Forced expiratory volume in one second at transplant in liter (FEV1)** |  |  | 0.854 (2) |
| - Missing | 115 | 22 |  |
| - Mean (SD) | 1.00 (0.68) | 1.0 (0.67) |  |
| - Range | 0.16 - 5.00 | 0.22 - 4.53 |  |
| **6 minute walk test** |  |  | 0.423 (2) |
| - Missing | 336 | 80 |  |
| - Mean (SD) | 327.94 (133.21) | 321.026 (136.90) |  |
| - Range | 0.000 - 750.00 | 24.000 - 750.00 |  |
| **Oxygen saturation in the arterial blood at transplant (SaO2)** |  |  | 0.368 (2) |
| - Missing | 1335 | 381 |  |
| - Mean (SD) | 84.36 (6.71) | 82.36 (7.84) |  |
| - Range | 68.000 - 96.00 | 69.00 - 93.00 |  |
| **Time between transplant and rejection in months** |  |  | 0.410 (2) |
| - Missing | 29 | 0 |  |
| - Mean (SD) | 58.81 (34.37) | 60.40 (31.10) |  |
| - Range | 3.00 - 144.00 | 3.00 - 136.00 |  |
| **Time between transplant and death in months** |  |  | 0.768 (2) |
| - Missing | 894 | 241 |  |
| - Mean (SD) | 41.88 (34.38) | 40.96 (31.79) |  |
| - Range | 0.00 - 135.00 | 3.00 - 121.00 |  |
| **Follow up time in months** |  |  | 0.530 (2) |
| - Missing | 63 | 2 |  |
| - Mean (SD) | 65.80 (35.33) | 67.05 (32.25) |  |
| - Range | 3.000 - 162.0 | 3.00 - 152.00 |  |
| **Number of visite** |  |  | 0.369 (2) |
| - Missing | 33 | 1 |  |
| - Mean (SD) | 10.83 (5.07) | 11.09 (4.70) |  |
| - Range | 2.00 - 26.00 | 2.00 - 22.00 |  |
| **Graft type** |  |  | 0.684 (1) |
| - Missing | 49 | 2 |  |
| - bi-pulmonary | 1154 (84.6%) | 335 (85.9%) |  |
| - cardio-pulmonary | 38 (2.8%) | 8 (2.1%) |  |
| - mono-pulmonary | 172 (12.6%) | 47 (12.1%) |  |
| **Lung type** |  |  | 0.995 (1) |
| - Missing | 55 | 8 |  |
| - bi-pulmonary | 1182 (87.0%) | 335 (87.2%) |  |
| - left | 79 (5.8%) | 22 (5.7%) |  |
| - right | 97 (7.1%) | 27 (7.0%) |  |
| **Number of graft** |  |  | 0.720 (2) |
| - Missing | 99 | 27 |  |
| - Mean (SD) | 1.05 (0.22) | 1.04 (0.21) |  |
| - Range | 1.00 - 3.00 | 1.00 - 3.00 |  |
| **Cause of register urgency** |  |  | 0.194 (1) |
| - Missing | 1251 | 350 |  |
| - extracorporeal circulation | 46 (28.4%) | 11 (26.2%) |  |
| - invasive ventilation | 33 (20.4%) | 14 (33.3%) |  |
| - invasive ventilation threat | 83 (51.2%) | 17 (40.5%) |  |
| **Preserving liquid** |  |  | **< 0.001** (1)(3) |
| - Missing | 434 | 68 |  |
| - Celsior | 198 (20.2%) | 20 (6.2%) |  |
| - Perfadex | 781 (79.8%) | 304 (93.8%) |  |
| **Ischemia in minutes** |  |  | 0.722 (2) |
| - Missing | 299 | 75 |  |
| - Mean (SD) | 372.59 (165.30) | 369.18 (77.54) |  |
| - Range | 105.00 - 4320.00 | 155.00 - 800.00 |  |
| **Lung reduction** |  |  | 0.030 (1)(4) |
| - Missing | 239 | 57 |  |
| - Atypical resections | 36 (3.1%) | 5 (1.5%) |  |
| - Lobectomy | 129 (11.0%) | 24 (7.2%) |  |
| - No | 1009 (85.9%) | 306 (91.3%) |  |
| **Surgical revision for hemostasis** |  |  | 0.807 (1) |
| - Missing | 75 | 7 |  |
| - No | 1179 (88.1%) | 341 (88.6%) |  |
| - Yes | 159 (11.9%) | 44 (11.4%) |  |
| **Primary graft dysfunction** |  |  | 0.071 (1) |
| - Missing | 1180 | 355 |  |
| - No | 185 (79.4%) | 34 (91.9%) |  |
| - Yes | 48 (20.6%) | 3 (8.1%) |  |
| **Induction treatment** |  |  | 0.005 (1)(4) |
| - Missing | 67 | 13 |  |
| - No | 401 (29.8%) | 85 (22.4%) |  |
| - Yes | 945 (70.2%) | 294 (77.6%) |  |
| **Thymoglobuline antibodies** |  |  | 0.046 (1)(4) |
| - Missing | 434 | 101 |  |
| - No | 472 (48.2%) | 121 (41.6%) |  |
| - Yes | 507 (51.8%) | 170 (58.4%) |  |
| **Anti CD3 antibody muronomab** |  |  | 0.913 (1) |
| - Missing | 477 | 117 |  |
| - No | 933 (99.7%) | 274 (99.6%) |  |
| - Yes | 3 (0.3%) | 1 (0.4%) |  |
| **Anti IL2 antibody daclizumab** |  |  | 0.257 (1) |
| - Missing | 440 | 99 |  |
| - No | 538 (55.3%) | 173 (59.0%) |  |
| - Yes | 435 (44.7%) | 120 (41.0%) |  |
| **Anti CD52 antibody alemtuzumab** |  |  | 0.915 (1) |
| - Missing | 476 | 116 |  |
| - No | 934 (99.7%) | 275 (99.6%) |  |
| - Yes | 3 (0.3%) | 1 (0.4%) |  |
| **Duration of post-transplant invasive mechanical ventilation (days)** |  |  | 0.489 (2) |
| - Missing | 158 | 40 |  |
| - Mean (SD) | 10.87 (30.93) | 9.64 (23.01) |  |
| - Range | 0.00 - 500.00 | 0.00 - 275.00 |  |
| **P/F ratio** |  |  | 0.789 (2) |
| - Missing | 1326 | 381 |  |
| - Mean (SD) | 266.32 (99.49) | 274.82 (92.57) |  |
| - Range | 101.00 - 400.00 | 130.00 - 400.00 |  |

1. Pearson’s Chi-squared test
2. Linear Model ANOVA
3. Significant
4. Nominally significant

*N.B. P/F ratio, ratio of arterial oxygen partial pressure (PaO2 in mmHg) to fractional inspired oxygen (FiO2); percentages excludes missing data.*

## **Supplementary Table 4**: Functional characteristics in GenCOLT and the rest of the COLT cohort for chronic obstructive pulmonary disease recipients.

|  | COLT (N=514) | GenCOLT (N=175) | p value |
| --- | --- | --- | --- |
| **Phenotype** |  |  | 0.886 (1) |
| - Missing | 86 | 6 |  |
| - Azithromycin-responsive allograft dysfunction (ARAD) | 8 (1.9%) | 4 (2.4%) |  |
| - Bronchiolitis obliterans syndrome (BOS) | 71 (16.6%) | 28 (16.6%) |  |
| - On wait | 10 (2.3%) | 2 (1.2%) |  |
| - Undefined | 12 (2.8%) | 5 (3.0%) |  |
| - Mixte | 6 (1.4%) | 3 (1.8%) |  |
| - Other | 101 (23.6%) | 44 (26.0%) |  |
| - Restrictive allograft syndrome (RAS) | 13 (3.0%) | 5 (3.0%) |  |
| - Non-CLAD with azithromycin | 91 (21.3%) | 26 (15.4%) |  |
| - Non-CLAD | 116 (27.1%) | 52 (30.8%) |  |
| **Center** |  |  | **< 0.001** (1)(3) |
| - Bordeaux | 87 (16.9%) | 21 (12.0%) |  |
| - Grenoble | 37 (7.2%) | 25 (14.3%) |  |
| - Le-Plessis-Robinson | 35 (6.8%) | 9 (5.1%) |  |
| - Lyon | 35 (6.8%) | 4 (2.3%) |  |
| - Marseille | 77 (15.0%) | 31 (17.7%) |  |
| - Nantes | 16 (3.1%) | 5 (2.9%) |  |
| - Paris-Bichat | 30 (5.8%) | 7 (4.0%) |  |
| - Paris-HEGP | 5 (1.0%) | 0 (0.0%) |  |
| - Strasbourg | 97 (18.9%) | 50 (28.6%) |  |
| - Suresnes | 66 (12.8%) | 9 (5.1%) |  |
| - Toulouse | 29 (5.6%) | 14 (8.0%) |  |
| **Forced expiratory volume in one second at transplant in liter (FEV1)** |  |  | 0.692 (2) |
| - Missing | 30 | 10 |  |
| - Mean (SD) | 0.73 (0.50) | 0.74 (0.50) |  |
| - Range | 0.16 - 5.00 | 0.22 - 3.60 |  |
| **Forced expiratory volume in one second at transplant (FEV1)** |  |  | 0.288 (2) |
| - Missing | 17 | 6 |  |
| - Mean (SD) | 23.88 (12.31) | 25.08 (14.05) |  |
| - Range | 3.35 - 102.30 | 11.00 - 102.30 |  |
| **6-minute walk test** |  |  | 0.524 (2) |
| - Missing | 98 | 26 |  |
| - Mean (SD) | 268.70 (118.43) | 275.99 (122.55) |  |
| - Range | 17.00 - 750.00 | 24.00 - 750.00 |  |

1. Pearson’s Chi-squared test
2. Linear Model ANOVA
3. Significant

*N.B. percentages excludes missing data.*

## **Supplementary Table 5**: Functional characteristics in GenCOLT and the rest of the COLT cohort for cystic fibrosis recipients

|  | COLT (N=413) | GenCOLT (N=84) | p value |
| --- | --- | --- | --- |
| **Phenotype** |  |  | 0.780 (1) |
| - Missing | 51 | 2 |  |
| - Azithromycin-responsive allograft dysfunction (ARAD) | 11 (3.0%) | 5 (6.1%) |  |
| - Bronchiolitis obliterans syndrome (BOS) | 43 (11.9%) | 10 (12.2%) |  |
| - On wait | 10 (2.8%) | 2 (2.4%) |  |
| - Undefined | 2 (0.6%) | 0 (0.0%) |  |
| - Mixte | 6 (1.7%) | 2 (2.4%) |  |
| - Other | 52 (14.3%) | 8 (9.8%) |  |
| - Restrictive allograft syndrome (RAS) | 5 (1.4%) | 1 (1.2%) |  |
| - Non-CLAD with azithromycin | 91 (25.1%) | 16 (19.5%) |  |
| - Non-CLAD | 142 (39.2%) | 38 (46.3%) |  |
| **Center** |  |  | **< 0.001** (1)(3) |
| - Bordeaux | 41 (9.9%) | 12 (14.3%) |  |
| - Grenoble | 7 (1.7%) | 1 (1.2%) |  |
| - Lyon | 42 (10.2%) | 4 (4.8%) |  |
| - Marseille | 70 (16.9%) | 21 (25.0%) |  |
| - Nantes | 46 (11.1%) | 14 (16.7%) |  |
| - Paris-HEGP | 28 (6.8%) | 4 (4.8%) |  |
| - Strasbourg | 35 (8.5%) | 18 (21.4%) |  |
| - Suresnes | 139 (33.7%) | 10 (11.9%) |  |
| - Toulouse | 5 (1.2%) | 0 (0.0%) |  |
| **Forced expiratory volume in one second at transplant in liter (FEV1)** |  |  | 0.771 (2) |
| - Missing | 21 | 3 |  |
| - Mean (SD) | 0.86 (0.37) | 0.84 (0.32) |  |
| - Range | 0.37 - 3.09 | 0.38 - 2.62 |  |
| **Forced expiratory volume in one second at transplant (FEV1)** |  |  | 0.337 (2) |
| - Missing | 10 | 1 |  |
| - Mean (SD) | 24.12 (6.99) | 23.32 (6.46) |  |
| - Range | 11.20 - 60.66 | 11.200 - 40.00 |  |
| **6-minute walk test** |  |  | 0.480 (2) |
| - Missing | 125 | 32 |  |
| - Mean (SD) | 419.73 (115.63) | 432.04 (114.89) |  |
| - Range | 0.00 - 730.00 | 125.00 - 625.00 |  |

1. Pearson’s Chi-squared test
2. Linear Model ANOVA
3. Significant

*N.B. percentages excludes missing data.*

## **Supplementary Table 6**: Functional characteristics in GenCOLT and the rest of the COLT cohort in interstitial lung disease recipients

|  | COLT (N=250) | GenCOLT (N=74) | p value |
| --- | --- | --- | --- |
| **Phenotype** |  |  | 0.945 (1) |
| - Missing | 33 | 2 |  |
| - Azithromycin-responsive allograft dysfunction (ARAD) | 6 (2.8%) | 1 (1.4%) |  |
| - Bronchiolitis obliterans syndrome (BOS) | 27 (12.4%) | 9 (12.5%) |  |
| - On wait | 3 (1.4%) | 1 (1.4%) |  |
| - Undefined | 6 (2.8%) | 2 (2.8%) |  |
| - Mixte | 6 (2.8%) | 3 (4.2%) |  |
| - Other def | 77 (35.5%) | 26 (36.1%) |  |
| - Restrictive allograft syndrome (RAS) | 11 (5.1%) | 1 (1.4%) |  |
| - Non-CLAD with azithromycin | 34 (15.7%) | 11 (15.3%) |  |
| - Non-CLAD | 47 (21.7%) | 18 (25.0%) |  |
| **Center** |  |  | 0.002 (1)(3) |
| - Bordeaux | 24 (9.6%) | 4 (5.4%) |  |
| - Grenoble | 7 (2.8%) | 6 (8.1%) |  |
| - Le-Plessis-Robinson | 21 (8.4%) | 4 (5.4%) |  |
| - Lyon | 19 (7.6%) | 3 (4.1%) |  |
| - Marseille | 52 (20.8%) | 27 (36.5%) |  |
| - Nantes | 8 (3.2%) | 3 (4.1%) |  |
| - Paris-Bichat | 24 (9.6%) | 2 (2.7%) |  |
| - Paris-HEGP | 7 (2.8%) | 0 (0.0%) |  |
| - Strasbourg | 26 (10.4%) | 12 (16.2%) |  |
| - Suresnes | 40 (16.0%) | 3 (4.1%) |  |
| - Toulouse | 22 (8.8%) | 10 (13.5%) |  |
| **Forced expiratory volume in one second at transplant in liter (FEV1)** |  |  | 0.235 (2) |
| - Missing | 23 | 5 |  |
| - Mean (SD) | 1.54 (0.62) | 1.64 (0.70) |  |
| - Range | 0.40 - 3.83 | 0.400 - 3.83 |  |
| **Forced expiratory volume in one second at transplant (FEV1)** |  |  | 0.569 (2) |
| - Missing | 12 | 5 |  |
| - Mean (SD) | 49.90 (18.44) | 51.36 (20.03) |  |
| - Range | 4.60 - 112.00 | 10.00 - 105.90 |  |
| **6-minute walk test** |  |  | 0.533 (2) |
| - Missing | 40 | 13 |  |
| - Mean (SD) | 312.95 (126.53) | 324.74 (141.10) |  |
| - Range | 30.00 - 597.00 | 30.00 - 560.00 |  |

1. Pearson’s Chi-squared test
2. Linear Model ANOVA
3. Nominally significant

*N.B. percentages excludes missing data.*

## **Supplementary Table 7**: Functional characteristics in GenCOLT and the rest of the COLT cohort in pulmonary hypertension recipients

|  | COLT (N=88) | GenCOLT (N=20) | p value |
| --- | --- | --- | --- |
| **Phenotype** |  |  | 0.875 (1) |
| - Missing | 16 | 1 |  |
| - Azithromycin-responsive allograft dysfunction (ARAD) | 1 (1.4%) | 0 (0.0%) |  |
| - Bronchiolitis obliterans syndrome (BOS) | 11 (15.3%) | 1 (5.3%) |  |
| - On wait | 1 (1.4%) | 0 (0.0%) |  |
| - Undefined | 1 (1.4%) | 0 (0.0%) |  |
| - Other | 18 (22.2%) | 4 (15.8%) |  |
| - Restrictive allograft syndrome (RAS) | 1 (1.4%) | 0 (0.0%) |  |
| - Non-CLAD with azithromycin | 15 (20.8%) | 5 (26.3%) |  |
| - Non-CLAD | 24 (33.3%) | 9 (47.4%) |  |
| **Center** |  |  | 0.528 (1) |
| - Bordeaux | 7 (8.0%) | 3 (15.0%) |  |
| - Grenoble | 2 (2.3%) | 1 (5.0%) |  |
| - Le-Plessis-Robinson | 55 (62.5%) | 9 (45.0%) |  |
| - Lyon | 9 (10.2%) | 1 (5.0%) |  |
| - Marseille | 2 (2.3%) | 0 (0.0%) |  |
| - Nantes | 3 (3.4%) | 1 (5.0%) |  |
| - Paris-HEGP | 1 (1.1%) | 0 (0.0%) |  |
| - Strasbourg | 8 (9.1%) | 5 (25.0%) |  |
| - Suresnes | 1 (1.1%) | 0 (0.0%) |  |
| **Forced expiratory volume in one second at transplant in liter (FEV1)** |  |  | 0.430 (2) |
| - Missing | 22 | 2 |  |
| - Mean (SD) | 2.38 (1.11) | 2.15 (1.00) |  |
| - Range | 0.47 - 5.00 | 0.470 - 4.53 |  |
| **Forced expiratory volume in one second at transplant (FEV1)** |  |  | 0.726 (2) |
| - Missing | 17 | 2 |  |
| - Mean (SD) | 73.47 (25.92) | 70.99 (29.62) |  |
| - Range | 4.28 - 122.00 | 14.00 - 110.40 |  |
| **6-minute walk test** |  |  | 0.256 (2) |
| - Missing | 31 | 3 |  |
| - Mean (SD) | 374.32 (118.60) | 336.35 (124.30) |  |
| - Range | 30.00 - 600.00 | 180.00 - 580.00 |  |

1. Pearson’s Chi-squared test
2. Linear Model ANOVA

*N.B. P/F ratio, ratio of arterial oxygen partial pressure (PaO2 in mmHg) to fractional inspired oxygen (FiO2); percentages excludes missing data.*


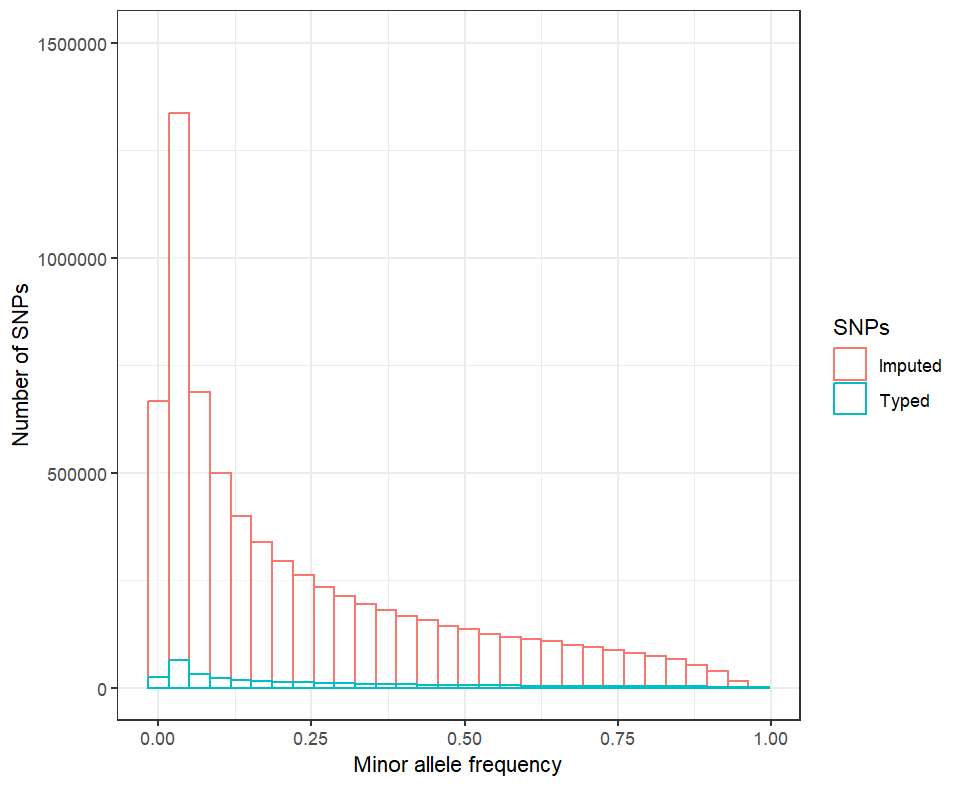


## **Supplementary Figure 1**: SNP enrichment through the SNP imputation process.

Before imputation in blue, we observed a lower number of SNPs (n=400,089). In red after imputation, we significantly increased the number of SNPs (n=7,337,433) available for subsequent association testing, especially those with a minor allele frequency (MAF) >1%.


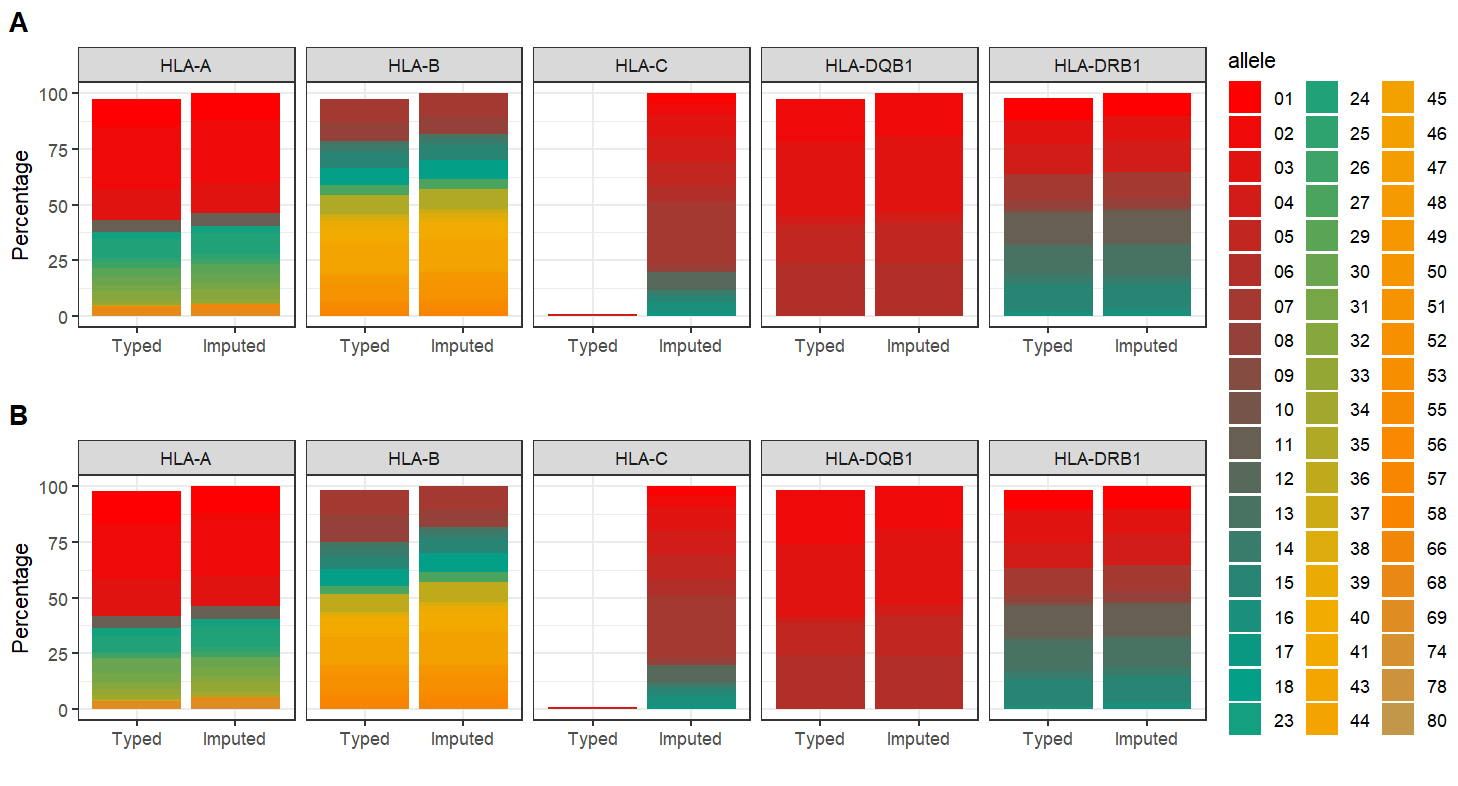


## **Supplementary Figure 2**: Distribution of *HLA* alleles in GenCOLT individuals according to typed vs. imputed *HLA-A*, *HLA-B*, *HLA-C*, *HLA*-*DRB1* and *HLA*-*DQB1* gene alleles.

We compared the *HLA* allele (in one-field resolution) distribution before and after imputation with HIBAG in GenCOLT donors (A) and recipients (B).


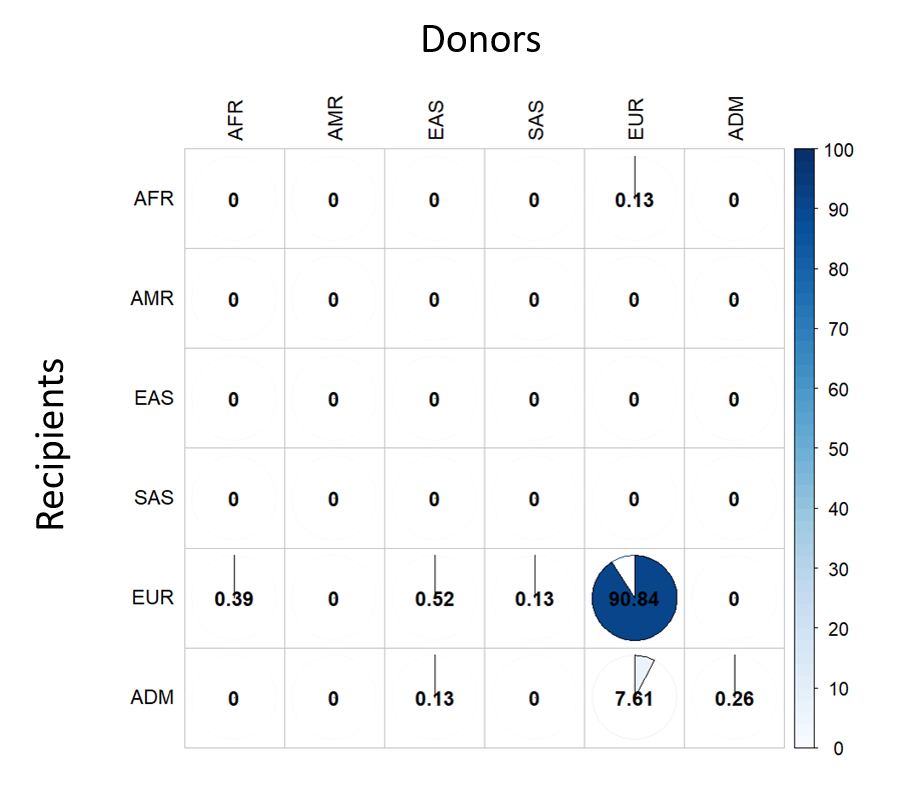


## **Supplementary Figure 3**: Genetic ancestry matching between donors and recipients from GenCOLT pairs.

We evaluated the distribution of genetic ancestry as defined with ADMIXTURE within pairs. Individuals were attributed to an ancestry group when the genetic ancestry proportion was ≥80% in one of the five ancestry reference groups; otherwise, individuals were classified as admixed (ADM).

*N.B. AFR, African; AMR, American; EAS, East Asian; EUR, European; SAS, South Asian.*
